# Quality of life and emotional distress among primary caregivers of children with intellectual disabilities: a comparative study in China

**DOI:** 10.3389/fpsyt.2026.1750568

**Published:** 2026-03-25

**Authors:** Yishu Dong, Jiao Yuan, Yaling Cheng, Yilin Wang, Weihua Pan

**Affiliations:** Department of Psychiatry, Qingdao Mental Health Center, Qingdao, China

**Keywords:** anxiety, caregivers, depression, intellectual disability, quality of life, WHOQOL-BREF

## Abstract

**Objective:**

To compare quality of life (QOL) and levels of anxiety and depression between primary caregivers of children with intellectual disabilities (ID) and caregivers of children without ID, and to identify sociodemographic and emotional correlates of caregivers’ QOL among those caring for children with ID.

**Methods:**

A total of 217 primary caregivers of children diagnosed with ID were recruited from Jinzhou Kangning Hospital between January and December 2023, and 141 caregivers of children without ID from the same region were recruited online. Caregivers completed the WHOQOL-BREF, and trained psychiatrists administered the Hamilton Anxiety Rating Scale (HAMA) and Hamilton Depression Rating Scale (HAMD-17). Group differences were examined using t-tests and ANOVA, and Pearson correlations assessed bivariate associations. Within the ID caregiver group, hierarchical multiple regression (Enter method) was performed to evaluate the impact of sociodemographic covariates, anxiety, and depression on WHOQOL-BREF domains. Model improvement was assessed using ΔR² and F-change tests.

**Results:**

Compared with the comparison group, caregivers of children with ID reported significantly lower QOL across all WHOQOL-BREF domains and significantly higher anxiety and depression. Within the ID caregiver group (N = 217), QOL was associated with caregiver kinship (relationship to the child), child characteristics, and family socioeconomic resources (e.g., income) in bivariate analyses. In multivariable models, adding anxiety and depression significantly increased explained variance in all domains in the ID caregiver group only (ΔR² = .034–.053). Anxiety independently predicted lower psychological well-being (PW) and social relationships (SR), whereas depression independently predicted lower environmental QOL (EN); neither symptom showed a unique association with physical capacity (PC) after adjustment. Several covariates remained significant in Model 1, including (selected examples) family income (PW, SR), profound ID (PW), caregiver kinship (SR), child gender (SR), and child age (EN).

**Conclusions:**

In the context of family caregiving in China, caregivers of children with ID experience poorer QOL and greater emotional distress than caregivers of children without ID. Anxiety and depression contribute additional explanatory power beyond sociodemographic factors, with domain-specific patterns of independent association. These findings support the need for accessible psychosocial support and family-centered services, and for longitudinal research to clarify temporal pathways.

## 1 Introduction

Intellectual disability (ID) is characterized by significant limitations in intellectual functioning and adaptive behavior, affecting conceptual, social, and practical domains, with onset during the developmental period (1). Beyond these functional challenges, children with ID often face psychiatric symptoms and physical comorbidities (2), which increase the support required in various settings such as home, school, and community. ID is prevalent, affecting approximately 1–3% of the global population (3), and in China, an estimated 1.75 million children live with ID, making it one of the most common childhood disabilities (4). In this context, families often provide long-term care and coordinate services, which closely links caregiver well-being to children’s participation, development, and overall family quality of life (5, 6).

According to Bowen’s (7) Family Systems Theory, a family functions as an interactive system rather than as isolated individuals. Most children with ID rely on primary caregivers for daily support, with around 71% of individuals with ID in the U.S. living with family members (8). Chronic stressors faced by caregivers include behavioral challenges (9), high demands (10), stigma (11), and financial pressure (12). These stressors can severely affect caregivers’ quality of life (QOL) and family functioning (13). Additionally, rehabilitation outcomes are closely tied to caregivers’ attitudes and their ability to manage the child’s impairments (6). Therefore, improving caregivers’ well-being is essential for enhancing the child’s development.

QOL refers to individuals’ subjective evaluations of their well-being, satisfaction, and fulfillment (14). It has become a key indicator in health research following the adoption of the bio-psychosocial model (15). Instruments like the World Health Organization Quality of Life-BREF (WHOQOL-BREF) and Family Quality of Life Scale are commonly used in ID research (16–21). Since the 1980s, QOL has been a significant focus in ID research (1).

In China, research on ID has predominantly focused on children’s rehabilitation and parents’ psychological well-being, with relatively less attention to the broader range of family caregivers (22). Unlike Western nuclear families, Chinese families are often highly interdependent and multigenerational, with grandparents frequently involved in caregiving and decision-making (23). Thus, limiting caregiver research to mothers and fathers may overlook critical sources of caregiving burden and support within the family system. Moreover, most studies examine correlates of caregiver QOL in isolation, focusing on factors like anxiety, depression, or income without considering the interrelated nature of these variables and their combined effect on QOL (24–26).

Guided by family systems theory and a biopsychosocial perspective, we conceptualize caregiver QOL as a multidimensional outcome shaped jointly by child characteristics (e.g., age, gender, degree of ID), family resources and family-structural indicators (e.g., income, only-child status, and caregiver kinship), and caregivers’ emotional distress (anxiety and depression). Although prior studies have documented poorer caregiver well-being in families of children with developmental disabilities, two gaps remain salient in the Chinese context (27). First, evidence is still limited on caregiver QOL when caregiving is often multigenerational and grandparents may serve as primary caregivers (22). Second, many studies report correlates in isolation, leaving it unclear whether anxiety and depression contribute to caregiver QOL independently of child- and family-level characteristics (24–26). Therefore, beyond comparing caregivers of children with and without ID, the present study integrates family-structural indicators (caregiver kinship and/or family structure, tested in alternative specifications) and emotional distress within a multivariable framework to test the incremental and domain-specific independent associations of anxiety and depression with QOL among ID caregivers in China.

The study addresses the following research questions:

1. Do primary caregivers of children with ID report lower WHOQOL-BREF domain scores and higher anxiety/depression than caregivers of children without ID?
2. Within the ID caregiver group, what are the bivariate associations between WHOQOL-BREF domain scores and sociodemographic characteristics, anxiety, and depression?
3. After adjusting for sociodemographic covariates, do anxiety and depression explain additional variance in WHOQOL-BREF domains (ΔR²), and do they show domain-specific independent associations?

Based on prior findings (28), parents of children with developmental disabilities tend to have lower QOL than parents of typically developing children, with gender, depression, anxiety, and stress exerting significant effects. Accordingly, this study hypothesises that:

1. Caregivers of children with ID will report lower QOL and higher anxiety and depression than caregivers of children without ID.
2. Within the ID caregiver group, higher anxiety and depression will be associated with lower QOL across domains.
3. Anxiety and depression will contribute incremental explanatory power for QOL domains beyond sociodemographic covariates, with potentially domain-specific independent effects.

## 2 Materials and methods

### 2.1 Participants

The study included primary caregivers of children aged 6–16 years (29) who met ICD-11 diagnostic criteria for ID (30) and received outpatient or inpatient care at Jinzhou Kangning Hospital between January and December 2023. The ID caregiver group comprised 217 participants. A comparison group of 141 caregivers of children without ID from the same region was recruited online. In the ID caregiver group, 231 caregivers initially responded and 14 withdrew, yielding 217 valid questionnaires. In the comparison group, 153 caregivers responded and 12 withdrew, yielding 141 valid questionnaires.

Eligible participants were immediate family members within three generations who lived with and provided primary care for the child. Only one caregiver per child was included. Although several family members may provide care, only one primary caregiver per child was selected. This decision was made to avoid multiple informants’ biases and ensure that the perspective of the primary caregiver—typically the most involved in daily care—was captured. Caregivers were eligible if they had no communication disorders or ID and no severe medical conditions that would interfere with participation; individuals who self-reported a physician-diagnosed psychiatric disorder were excluded. Participation was voluntary, and written informed consent was obtained from all participants, who could withdraw at any time. Confidentiality and data anonymity were ensured. Each participant received a kitchen utensil worth 20 RMB as compensation.

Because the ID caregiver group was recruited in a hospital setting whereas the comparison group was recruited online, the two samples may differ in unmeasured characteristics (e.g., service access, health status, or willingness to participate), which should be considered when interpreting between-group differences.

### 2.2 Materials

#### 2.2.1 Assessment instruments

Four instruments were used: a Sociodemographic Questionnaire, the World Health Organization Quality of Life-BREF (WHOQOL-BREF) (31), the Hamilton Anxiety Rating Scale (HAMA) (32), and the 17-item Hamilton Depression Rating Scale (HAMD-17) (33), to assess quality of life (QOL), anxiety, and depression in both groups.

##### 2.2.1.1 Sociodemographic questionnaire

A self-developed questionnaire, designed and reviewed by two psychiatrists with over two years of clinical experience, was finalized between January and February 2023. It collected information on:

1. Caregiver kinship (father, mother, grandfather, grandmother);
2. Child’s gender (boy or girl);
3. Child’s age (6–8, 9–11, 12–14, 15–16 years);
4. Only-child status (yes/no);
5. Degree of ID (mild, moderate, severe, or profound);
6. Family annual per-capita income (<20000; 20000–40000; 40000–60000; 60000–80000; >80000 RMB);
7. Family structure (nuclear, stem, intergenerational, or single-parent family).

##### 2.2.1.2 World Health Organization Quality of Life-BREF

The WHOQOL-BREF (31) is a 24-item self-report scale assessing four domains: physical capacity (PC), psychological well-being (PW), social relationships (SR), and environment (EN). Items are rated on a 5-point scale, with higher scores reflecting better QOL. The instrument demonstrates reliability coefficients between 0.42 and 0.93 and structural validity above 0.90 (34). Compared with the longer WHOQOL-100, the WHOQOL-BREF provides comparable psychometric validity while being faster to administer, making it suitable for clinical and large-scale research. The WHOQOL-BREF was selected for this study because it is a widely recognized and reliable instrument that assesses the caregiver’s own perceived quality of life across multiple domains.

In the current study, internal consistency of the WHOQOL-BREF domains in the full sample (N = 358) was good: PC α = .927, PW α = .890, SR α = .815, and EN α = .920. Reliability estimates were comparable when calculated within the ID caregiver group (N = 217).

##### 2.2.1.3 Hamilton anxiety scale

The HAMA (32) is a clinician-rated scale widely used to assess anxiety, with reliability coefficients of 0.83–1.00 and a validity coefficient of 0.36. It comprises 14 items rated from None to Very Severe. In this study, HAMA total scores were categorized as follows: <14 = no/minimal anxiety, 14–20 = mild anxiety, 21–28 = moderate anxiety, and ≥29 = severe anxiety.

##### 2.2.1.4 Hamilton depression scale (HAMD-17)

The HAMD-17 (33) is a clinician-rated measure of depression with reliability coefficients of 0.88–0.99 and validity of 0.92 (32). It assesses 17 items on a 5-point scale from None to Very Severe. In this study, HAMD-17 total scores were categorized as follows: <7 = no/minimal depression, 7–13 = mild depression, 14–19 = moderate depression, and ≥20 = severe depression (35). Compared with self-report tools, the HAMD-17 provides a more objective evaluation of depressive severity (36).

### 2.3 Statistical analysis

Data were analyzed in SPSS 27.0 (Chinese version), with two-tailed significance set at p <.05. Descriptive statistics (frequencies, means, and standard deviations) summarized sample characteristics. Between-group differences in WHOQOL-BREF domain scores and emotional distress (HAMA/HAMD-17) were tested using independent-samples t-tests and one-way ANOVA as appropriate; effect sizes were reported as Cohen’s d and partial eta squared (η²). Assumptions for parametric analyses (distributional shape and homogeneity of variance) were iinspected (histograms/Q–Q plots and Levene’s test) prior to hypothesis testing. When distributional assumptions were questionable, non-parametric robustness checks were conducted for selected comparisons/associations (Mann–Whitney U tests for between-group comparisons; Spearman correlations for associations), yielding the same pattern of conclusions as the parametric analyses.

Within the ID caregiver group (N = 217), hierarchical multiple regression analyses (Enter method) were conducted separately for each WHOQOL-BREF domain (PC, PW, SR, EN). Sociodemographic covariates (dummy-coded with a reference category) were entered in Block 1, followed by anxiety and depression entered simultaneously in Block 2. Model improvement was evaluated using ΔR² and the F-change test, and standardized coefficients (β) were reported. To mitigate multicollinearity and conceptual overlap, family structure was not entered simultaneously with caregiver kinship; multicollinearity diagnostics were acceptable in the final models (VIFs < 2). Sensitivity analyses compared alternative specifications (caregiver kinship *vs*. family structure) to assess the robustness of key inferences.

Additionally, the assumptions of residual normality, homoscedasticity, and independence of errors were examined. Residuals were assessed for normality using Q-Q plots and the Shapiro-Wilk test. Homoscedasticity was evaluated using scatterplots of residuals against fitted values, and no patterns indicating heteroscedasticity were observed. The Durbin-Watson statistic was used to assess the independence of errors, and values between 1.5 and 2.5 suggested no significant autocorrelation. These diagnostics confirmed that the regression models met the necessary assumptions for validity.

### 2.4 Procedure

Data were collected via the WHOQOL-BREF, HAMA, HAMD-17, and Sociodemographic Questionnaire. A total of 384 questionnaires were distributed through the Medical Education Department of Jinzhou Kangning Hospital. ID caregiver group caregivers were asked to bring their child’s medical records, ID, and household registration, while comparison caregivers provided ID and registration documents.

At the study site, two trained psychiatrists introduced the study in neutral terms, explaining that the project aimed to understand caregivers’ quality of life and emotional well-being (anxiety and depressive symptoms) and their relationships with child- and family-related characteristics. To minimize expectancy effects, the consent discussion did not emphasize any assumed direction of group differences. All participants provided written informed consent before enrollment and were reminded that participation was voluntary and that they could withdraw at any time without consequences.

The HAMA and HAMD-17 were administered by two trained psychiatrists according to standardized procedures. Prior to data collection, both raters completed training and calibration sessions to harmonize scoring criteria. Throughout the study, regular consensus meetings were held to ensure scoring consistency and address any scoring uncertainties to minimize rater drift. Formal inter-rater reliability indices (e.g., ICC) were not estimated; however, rater training, calibration, and ongoing consensus procedures were used to maintain scoring consistency.

### 2.5 Ethics statement

Ethical approval was obtained from the Institutional Review Board (IRB)/Ethics Committee of Jinzhou Kangning Hospital (Jinzhou, Liaoning, China). The study complied with the principles of the 1964 Declaration of Helsinki. Written informed consent was secured from all participants, ensuring voluntary participation, privacy, and data confidentiality. Personal information was anonymised and used solely for academic research.

## 3 Results

### 3.1 Comparison of sociodemographic data between the ID caregiver group and the comparison group

A total of 358 participants were included: 217 in the ID caregiver group and 141 in the comparison group. The ID caregiver group consisted of 43 fathers, 127 mothers, 12 grandfathers, and 35 grandmothers; the comparison group comprised 36 fathers, 81 mothers, 10 grandfathers, and 14 grandmothers. No significant between-group differences were observed in the sociodemographic characteristics shown in **Table 1** (all *p* >.05).

**Table 1:** Comparison of sociodemographic data between the ID caregiver group and the comparison group.

Characteristics	ID caregiver group(N = 217)	Comparison group(N = 141)	χ2	*p*-value
Caregiver kinship				
Father	43	36	4.022	.259
Mother	127	81		
Grandfather	12	10		
Grandmother	35	14		
Child’s gender				
Boy	109	72	0.024	.878
Girl	108	69		
Age of the child (years)				
6-8	69	45	0.603	.896
9-11	83	54		
12-14	42	24		
15-16	23	18		
Only-child status				
Yes	76	48	0.036	.849
No	141	93		
Degree of intellectual disability in children				
Mild	74	–	NA	NA
Moderate	72	–		
Severe	46	–		
Profound	25	–		
Family annual per-capita income (RMB)				
<20000	64	42	4.52	.340
20000-40000	75	52		
40000-60000	45	32		
60000-80000	15	11		
>80000	18	4		
Family structure				
Nuclear family	85	62	1.711	.635
Stem family	71	46		
Intergenerational family	38	18		
Single-parent family	23	15		

Degree of ID was assessed in the ID caregiver group only; therefore, no between-group test was conducted.

### 3.2 Evaluation and comparison of the QOL of the two groups

Independent-samples t tests showed that primary caregivers of children with ID had significantly lower QOL scores across all domains than those of children without ID. Specifically, PC (*t*(356) = −29.434, 95% CI [−3.498, −2.867], *p* <.001, *d* = −3.184), PW (*t*(356) = −6.166, 95% CI [−0.884, −0.449], *p* <.001, *d* = −0.667), SR (*t*(356) = −6.606, 95% CI [−0.932, −0.496], *p* <.001, *d* = −0.715), and EN (*t*(356) = −5.828, 95% CI [−0.847, −0.413], *p* <.001, *d* = −0.630) were all significantly reduced in the ID caregiver group (**Table 2**).

**Table 2:** Comparison of QOL between the ID caregiver group and the comparison group.

Domain	ID caregiver group(N = 217) (Mean ± SD)	Comparison group(N = 141) (Mean ± SD)	t-value	*p*-value
PC	10.39 ± 1.34	14.19 ± 0.93	-29.434	<0.001
PW	10.86 ± 2.85	12.82 ± 3.07	-6.166	<0.001
SR	9.95 ± 4.30	13.17 ± 4.83	-6.606	<0.001
EN	10.49 ± 4.03	13.22 ± 4.76	-5.828	<0.001

PC, physical capacity; PW, psychological well-being; SR, social relationships; EN, environment.

### 3.3 Impact of sociodemographic data on the QOL of primary caregivers of children with ID

ANOVA showed a significant main effect of caregiver kinship on QOL scores in the SR domain among caregivers of children with ID (*F*(3,213) = 2.777, 95% CI [0.000, 0.088], *p* = .042, η² = 0.038), but not in PC, PW, or EN domains. Bonferroni *post hoc* tests indicated that fathers scored higher than grandfathers in the SR domain (*p* <.05).

T-tests revealed gender-related differences in the SR (*t*(215) = −4.160, 95% CI [−0.836, −0.293], *p* <.001, *d* = −0.565) and EN (*t*(215) = −2.147, 95% CI [−0.559, −0.024], *p* = .033, *d* = −0.292) domains, with higher scores among caregivers of girls.

Children’s age significantly affected caregivers’ QOL in PC (*F*(3,213) = 3.469, *p* = .017, η² = 0.047), PW (*F*(3,213) = 4.088, *p* = .008, η² = 0.054), SR (*F*(3,213) = 7.938, *p* <.001, η² = 0.101), and EN (*F*(3,213) = 17.982, *p* <.001, η² = 0.202) domains. Caregivers of children aged 12–14 scored higher than those caring for younger children (*p* <.05).

QOL differed by only-child status in the PW (*t*(215) = −3.652, *p* <.001, *d* = −0.520), SR (*t*(215) = −2.503, *p* = .013, *d* = −0.356), and EN (*t*(215) = −2.912, *p* = .004, *d* = −0.414) domains; caregivers of non–only children scored higher (*p* <.05).

The degree of ID significantly influenced QOL across all domains—PC (*F*(3,213) = 3.793, *p* = .011, η² = 0.051), PW (*F*(3,213) = 5.306, *p* = .002, η² = 0.070), SR (*F*(3,213) = 3.078, *p* = .028, η² = 0.042), and EN (F(3,213) = 2.950, *p* = .034, η² = 0.040). Caregivers of children with mild ID had higher PW and EN scores than those caring for children with severe or profound ID (*p* <.05).

Family income also showed significant effects in all domains—PC (*F*(4,212) = 4.252, *p* = .002, η² = 0.074), PW (*F*(4,212) = 8.477, *p* <.001, η² = 0.138), SR (*F*(4,212) = 8.255, *p* <.001, η² = 0.135), and EN (*F*(4,212) = 5.859, *p* <.001, η² = 0.100). Caregivers from higher-income families (>80,000 RMB) reported significantly higher QOL than those from lower-income families (<40,000 RMB) (*p* <.05).

Family structure significantly affected QOL in PC (*F*(3,213) = 3.522, *p* = .016, η² = 0.047), SR (*F*(3,213) = 3.538, *p* = .016, η² = 0.047), and EN (*F*(3,213) = 3.770, *p* = .011, η² = 0.050), but not in PW. Bonferroni tests indicated that caregivers from nuclear families scored higher in SR and EN than those from intergenerational families (*p* <.05) (**Table 3**).

**Table 3:** QOL of primary caregivers of children with ID with different sociodemographic data.

Characteristics	PC (Mean ± SD)	PW (Mean ± SD)	SR (Mean ± SD)	EN (Mean ± SD)
Caregiver kinship				
Father	10.58 ± 1.32	11.07 ± 2.92	10.79 ± 4.26	10.42 ± 3.82
Mother	10.45 ± 1.38	10.83 ± 2.71	10.17 ± 4.16	10.94 ± 3.96
Grandfather	10.24 ± 1.28	11.78 ± 3.17	9.78 ± 5.28	10.79 ± 4.65
Grandmother	9.96 ± 1.21	10.36 ± 3.17	8.15 ± 4.16	8.84 ± 4.09
Child’s gender				
Boy	10.29 ± 1.26	10.63 ± 2.84	8.78 ± 3.97	9.91 ± 4.04
Girl	10.48 ± 1.42	11.09 ± 2.86	11.12 ± 4.31	11.07 ± 3.96
Age of the child (years)				
6-8	10.20 ± 1.21	10.51 ± 2.78	9.24 ± 4.21	9.58 ± 3.76
9-11	10.20 ± 1.29	10.35 ± 2.66	9.09 ± 4.10	9.13 ± 3.61
12-14	10.88 ± 1.59	11.87 ± 3.08	12.63 ± 4.31	13.67 ± 3.46
15-16	10.71 ± 1.18	11.86 ± 2.77	10.26 ± 3.33	12.30 ± 3.59
Only-child status				
Yes	10.19 ± 1.17	9.92 ± 2.39	8.96 ± 4.22	9.42 ± 4.09
No	10.49 ± 1.42	11.36 ± 2.95	10.48 ± 4.26	11.06 ± 3.90
Degree of intellectual disability in children				
Mild	10.62 ± 1.47	11.55 ± 3.08	10.50 ± 4.46	11.20 ± 4.14
Moderate	10.57 ± 1.31	11.17 ± 2.66	10.52 ± 4.69	10.76 ± 4.18
Severe	9.99 ± 1.18	9.99 ± 2.52	9.22 ± 3.44	9.90 ± 3.46
Profound	9.90 ± 1.05	9.52 ± 2.51	8.00 ± 3.38	8.68 ± 3.78
Family annual per-capita income (RMB)				
<20000	10.25 ± 1.11	10.21 ± 2.25	8.48 ± 3.12	10.06 ± 3.35
20000-40000	10.13 ± 1.09	10.20 ± 2.92	9.44 ± 4.71	9.47 ± 4.26
40000-60000	10.41 ± 1.50	11.29 ± 2.89	10.46 ± 4.41	10.79 ± 4.02
60000-80000	10.93 ± 1.94	12.22 ± 2.90	12.71 ± 3.38	12.67 ± 4.05
>80000	11.40 ± 1.60	13.70 ± 2.10	13.70 ± 3.29	13.69 ± 3.13
Family structure				
Nuclear family	10.57 ± 1.40	10.93 ± 2.69	10.76 ± 4.17	11.13 ± 4.14
Stem family	10.56 ± 1.37	11.43 ± 2.89	10.22 ± 4.21	10.96 ± 3.97
Intergenerational family	10.02 ± 1.21	10.46 ± 3.12	8.42 ± 4.34	9.01 ± 4.05
Single-parent family	9.79 ± 0.96	9.51 ± 2.44	8.64 ± 4.25	9.11 ± 2.88

PC, physical capacity; PW, psychological well-being; SR, social relationships; EN, environment.

### 3.4 Study of the relationship between sociodemographic data and the QOL of primary caregivers of children with ID

Pearson’s correlation analysis revealed significant associations between caregivers’ QOL and several sociodemographic factors, including caregiver kinship, child’s gender, only-child status, and family structure (*p* <.05). QOL was positively correlated with the child’s age and family income, but negatively correlated with the child’s degree of ID (*p* <.05) (**Table 4**).

**Table 4:** Correlation between sociodemographic data and QOL scores of primary caregivers of children with ID.

Characteristics	PC	PW	SR	EN
Caregiver kinship	-.121	-.009	-.146*	-.071
Child’s gender	.071	.080	.273**	.145*
Age of the child (years)	.173*	.189**	.197**	.333**
Only-child status	.108	.242**	.168*	.195**
Degree of intellectual disability in children	-.206*	-.256**	-.185**	-.194**
Family annual per-capita income (RMB)	.232**	.346**	.361**	.267**
Family structure	-.197**	-.135*	-.202**	-.200**

PC, physical capacity; PW, psychological well-being; SR, social relationships; EN, environment. *p <.05, **p <.01.

### 3.5 Comparison of anxiety scores between the two groups

Independent-samples t test results showed significantly higher anxiety scores in the ID caregiver group compared with the comparison group (*p* <.05) (**Table 5**).

**Table 5:** Comparison of anxiety scores between study and comparison groups.

Groups	level	N (%)	Mean ± SD	t-value	*p*-value
ID caregiver group(N = 217)	No/minimal	136(62.7%)	12.22 ± 6.45	5.893	<.001
	Mild	53(24.4%)			
	Moderate	25(11.5%)			
	Severe	3(1.4%)			
Comparison group(N = 141)	No/minimal	122(86.6%)	8.61 ± 4.15		
	Mild	15(10.6%)			
	Moderate	4(2.8%)			
	Severe	–			

### 3.6 Correlation between anxiety and QOL of primary caregivers of children with ID

Anxiety scores among caregivers of children with ID were significantly and negatively correlated with all QOL domains (*p* <.05) (**Table 6**).

**Table 6:** Correlation between anxiety and QOL of primary caregivers of children with ID.

Characteristics	PC	PW	SR	EN	Anxiety
PC	1				
PW	.272**	1			
SR	.220**	.436**	1		
EN	.240**	.417**	.512**	1	
Anxiety	-.179**	-.261**	-.286**	-.168*	1

PC, physical capacity; PW, psychological well-being; SR, social relationships; EN, environment. *p <.05, **p <.01.

### 3.7 Comparison of depression scores between the two groups

Independent-samples t test results showed significantly higher depression scores in the ID caregiver group than in the comparison group (*p* <.05) (**Table 7**).

**Table 7:** Comparison of depression score between study and comparison groups.

Groups	level	N (%)	Mean ± SD	t-value	*p*-value
ID caregiver group(N = 217)	No/minimal	102(47.0%)	8.19 ± 4.85	10.037	<.001
	Mild	69(31.8%)			
	Moderate	46(21.2%)			
	Severe	–			
Comparison group(N = 141)	No/minimal	123(87.2%)	4.62 ± 1.61		
	Mild	18(12.8%)			
	Moderate	–			
	Severe	–			

### 3.8 Correlation between depression and QOL of primary caregivers of children with ID

Depression scores among caregivers of children with ID were significantly and negatively correlated with all QOL domains (*p* <.05) (**Table 8**).

**Table 8:** Correlation between depression and QOL of primary caregivers of children with ID.

Characteristics	PC	PW	SR	EN	Depression
PC	1				
PW	.272**	1			
SR	.220**	.436**	1		
EN	.240**	.417**	.512**	1	
Depression	-.171*	-.198**	-.219**	-.194**	1

PC, physical capacity; PW, psychological well-being; SR, social relationships; EN, environment. *p <.05, **p <.01.

### 3.9 Regression models predicting QOL domain scores (Enter method)

Hierarchical multiple regression analyses (Enter method) were conducted within the ID caregiver group (N = 217) to examine whether anxiety and depression were independently associated with WHOQOL-BREF domain scores. Four separate models were fitted with each WHOQOL-BREF domain as the dependent variable (PC, PW, SR, EN). In Model 1, sociodemographic covariates were entered simultaneously, including caregiver kinship, child gender, child age group, only-child status, degree of ID, and family annual per-capita income (all categorical predictors dummy-coded with appropriate reference categories). Because multiple categorical predictors were dummy-coded, Model 1 contained 15 predictors (excluding the intercept). In Model 2, anxiety (HAMA total score) and depression (HAMD-17 total score) were added simultaneously, yielding 17 predictors in total. Model improvement was evaluated using ΔR² and the F-change test. To ensure full transparency, **Tables 9A**, **9B**, **9C**, **9D** presents coefficients for all predictors entered in each model, regardless of statistical significance.

**Table 9A:** Hierarchical regression predicting PC.

Predictor	Model 1 B (SE)	β	p-value	Model 2 B (SE)	β	p-value
Intercept	10.148 (0.488)	—	<.001	10.827 (0.538)	—	<.001
Caregiver kinship (ref: Father)						
Mother	−0.121 (0.237)	−0.045	0.609	−0.123 (0.233)	−0.045	0.6
Grandfather	−0.410 (0.307)	−0.113	0.184	−0.314 (0.305)	−0.086	0.304
Grandmother	−0.178 (0.434)	−0.030	0.683	−0.259 (0.429)	−0.044	0.548
Child age (ref: 6–8)						
9–11	0.003 (0.217)	0.001	0.987	0.028 (0.214)	0.01	0.897
12–14	0.247 (0.280)	0.073	0.379	0.218 (0.279)	0.064	0.436
15–16	0.227 (0.330)	0.052	0.492	0.288 (0.326)	0.066	0.378
Degree of ID (ref: Mild)						
Moderate	0.139 (0.233)	0.049	0.551	0.185 (0.232)	0.065	0.427
Severe	−0.342 (0.258)	−0.104	0.187	−0.336 (0.255)	−0.102	0.189
Profound	−0.430 (0.317)	−0.103	0.177	−0.303 (0.316)	−0.072	0.338
Income (ref: <20000 RMB)						
20000–40000	−0.139 (0.226)	−0.049	0.54	−0.280 (0.229)	−0.100	0.222
40000–60000	−0.001 (0.270)	0	0.996	−0.260 (0.283)	−0.079	0.359
60000–80000	0.375 (0.401)	0.071	0.351	0.320 (0.397)	0.061	0.421
>80000	0.796 (0.386)	0.164	0.041	0.558 (0.391)	0.115	0.155
Child gender (ref: Boy)						
Girl	0.176 (0.188)	0.066	0.349	0.162 (0.185)	0.06	0.384
Only child (ref: No)						
Yes	0.048 (0.197)	0.017	0.806	0.068 (0.194)	0.024	0.726
Emotional distress (Model 2 only)						
Anxiety (HAMA)	—	—	—	−0.025 (0.016)	−0.119	0.118
Depression (HAMD-17)	—	—	—	−0.023 (0.013)	−0.126	0.087

**Table 9B:** Hierarchical regression predicting PW.

Predictor	Model 1 B (SE)	β	p-value	Model 2 B (SE)	β	p-value
Intercept	9.084 (0.986)	—	<.001	10.858 (1.074)	—	<.001
Caregiver kinship (ref: Father)						
Mother	−0.132 (0.479)	−0.023	0.783	−0.133 (0.466)	−0.023	0.776
Grandfather	−0.057 (0.621)	−0.007	0.927	0.186 (0.608)	0.024	0.76
Grandmother	1.263 (0.878)	0.102	0.152	1.040 (0.857)	0.084	0.226
Child age (ref: 6–8)						
9–11	−0.227 (0.438)	−0.039	0.604	−0.159 (0.426)	−0.027	0.709
12–14	0.237 (0.567)	0.033	0.676	0.185 (0.557)	0.026	0.74
15–16	0.480 (0.666)	0.052	0.473	0.651 (0.651)	0.07	0.318
Degree of ID (ref: Mild)						
Moderate	−0.033 (0.472)	−0.006	0.944	0.066 (0.462)	0.011	0.886
Severe	−0.844 (0.522)	−0.121	0.107	−0.840 (0.509)	−0.121	0.1
Profound	−1.362 (0.641)	−0.153	0.035	−1.031 (0.630)	−0.116	0.104
Income (ref: <20000 RMB)						
20000–40000	−0.090 (0.456)	−0.015	0.845	−0.469 (0.457)	−0.078	0.306
40000–60000	0.703 (0.546)	0.1	0.199	0.018 (0.564)	0.003	0.975
60000–80000	1.134 (0.811)	0.101	0.163	0.969 (0.792)	0.086	0.223
>80000	2.617 (0.781)	0.254	<.001	1.982 (0.781)	0.192	0.012
Child gender (ref: Boy)						
Girl	0.298 (0.379)	0.052	0.433	0.266 (0.369)	0.047	0.473
Only child (ref: No)						
Yes	0.769 (0.398)	0.129	0.055	0.817 (0.388)	0.137	0.036
Emotional distress (Model 2 only)						
Anxiety (HAMA)	—	—	—	−0.072 (0.031)	−0.164	0.022
Depression (HAMD-17)	—	—	—	−0.053 (0.027)	−0.135	0.052

**Table 9C:** Hierarchical regression predicting SR.

Predictor	Model 1 B (SE)	β	p-value	Model 2 B (SE)	β	p-value
Intercept	5.569 (1.429)	—	<.001	8.308 (1.549)	—	<.001
Caregiver kinship (ref: Father)						
Mother	−0.710 (0.693)	−0.082	0.307	−0.711 (0.672)	−0.082	0.291
Grandfather	−1.958 (0.900)	−0.168	0.031	−1.586 (0.877)	−0.136	0.072
Grandmother	−0.263 (1.272)	−0.014	0.836	−0.614 (1.236)	−0.033	0.62
Child age (ref: 6–8)						
9–11	−0.202 (0.634)	−0.023	0.751	−0.095 (0.615)	−0.011	0.878
12–14	1.855 (0.821)	0.171	0.025	1.787 (0.803)	0.165	0.027
15–16	0.219 (0.965)	0.016	0.821	0.490 (0.938)	0.035	0.602
Degree of ID (ref: Mild)						
Moderate	0.663 (0.683)	0.073	0.333	0.807 (0.667)	0.089	0.227
Severe	−0.106 (0.756)	−0.010	0.888	−0.108 (0.734)	−0.010	0.883
Profound	−1.187 (0.929)	−0.088	0.203	−0.676 (0.909)	−0.050	0.458
Income (ref: <20000 RMB)						
20000–40000	1.059 (0.661)	0.118	0.11	0.468 (0.658)	0.052	0.478
40000–60000	1.404 (0.791)	0.133	0.077	0.341 (0.813)	0.032	0.676
60000–80000	2.265 (1.174)	0.134	0.055	1.999 (1.142)	0.118	0.082
>80000	3.966 (1.131)	0.255	<.001	2.976 (1.126)	0.191	0.009
Child gender (ref: Boy)						
Girl	2.232 (0.550)	0.26	<.001	2.184 (0.533)	0.255	<.001
Only child (ref: No)						
Yes	0.166 (0.577)	0.018	0.774	0.237 (0.559)	0.026	0.672
Emotional distress (Model 2 only)						
Anxiety (HAMA)	—	—	—	−0.116 (0.045)	−0.174	0.011
Depression (HAMD-17)	—	—	—	−0.077 (0.039)	−0.131	0.048

**Table 9D:** Hierarchical regression predicting EN.

Predictor	Model 1 B (SE)	β	p-value	Model 2 B (SE)	β	p-value
Intercept	7.339 (1.337)	—	<.001	9.383 (1.465)	—	<.001
Caregiver kinship (ref: Father)						
Mother	0.725 (0.649)	0.089	0.265	0.713 (0.635)	0.087	0.263
Grandfather	−0.463 (0.842)	−0.042	0.583	−0.158 (0.829)	−0.014	0.849
Grandmother	1.396 (1.191)	0.079	0.243	1.183 (1.169)	0.067	0.313
Child age (ref: 6–8)						
9–11	−0.619 (0.594)	−0.075	0.298	−0.559 (0.582)	−0.068	0.338
12–14	3.260 (0.769)	0.32	<.001	3.098 (0.759)	0.304	<.001
15–16	2.180 (0.903)	0.167	0.017	2.332 (0.888)	0.178	0.009
Degree of ID (ref: Mild)						
Moderate	−0.208 (0.639)	−0.024	0.745	−0.020 (0.631)	−0.002	0.974
Severe	−0.239 (0.707)	−0.024	0.736	−0.183 (0.694)	−0.019	0.792
Profound	−1.300 (0.869)	−0.103	0.136	−0.916 (0.860)	−0.073	0.288
Income (ref: <20000 RMB)						
20000–40000	−0.433 (0.618)	−0.051	0.485	−0.833 (0.623)	−0.098	0.183
40000–60000	0.498 (0.740)	0.05	0.502	−0.250 (0.769)	−0.025	0.745
60000–80000	0.310 (1.099)	0.02	0.778	0.206 (1.081)	0.013	0.849
>80000	1.202 (1.059)	0.082	0.258	0.534 (1.065)	0.037	0.617
Child gender (ref: Boy)						
Girl	0.898 (0.514)	0.112	0.082	0.840 (0.504)	0.104	0.097
Only child (ref: No)						
Yes	0.574 (0.540)	0.068	0.289	0.648 (0.529)	0.077	0.222
Emotional distress (Model 2 only)						
Anxiety (HAMA)	—	—	—	−0.051 (0.043)	−0.082	0.233
Depression (HAMD-17)	—	—	—	−0.092 (0.037)	−0.167	0.013

All categorical predictors were dummy-coded with the following reference categories: Father (caregiver kinship), Boy (child gender), age 6–8 years, non–only child, mild ID, and annual per-capita income <20000 RMB. Model 1 includes sociodemographic covariates (15 predictors); Model 2 adds anxiety (HAMA) and depression (HAMD-17) simultaneously (17 predictors total). B = unstandardized coefficient; SE = standard error; β = standardized coefficient. ΔR² and the F-change test evaluate improvement from Model 1 to Model 2. Multicollinearity diagnostics were acceptable (VIFs < 2).

In the PC domain, Model 1 was significant, *F*(15, 201) = 1.901, *p* = .025, explaining 12.4% of the variance (R² = .124; adjusted R² = .059). Adding anxiety and depression significantly improved model fit (ΔR² = .034; *F*-change(2, 199) = 4.002, *p* = .020), and Model 2 remained significant, *F*(17, 199) = 2.198, *p* = .005 (R² = .158; adjusted R² = .086). However, neither anxiety (*p* = .118) nor depression (*p* = .087) showed a statistically significant unique association with PC in the final model (**Table 9A**).

In the PW domain, Model 1 was significant, *F*(15, 201) = 3.484, *p* <.001, explaining 20.6% of the variance (R² = .206; adjusted R² = .147). After adding anxiety and depression, Model 2 explained 25.7% of the variance (R² = .257; adjusted R² = .193), representing a significant increment (ΔR² = .050; *F*-change(2, 199) = 6.742, *p* = .001), and Model 2 remained significant, *F*(17, 199) = 4.043, *p* <.001. In the final model, anxiety was a significant negative predictor of PW (B = −0.072, β = −0.164, *p* = .022), whereas depression showed a marginal association (B = −0.053, β = −0.135, *p* = .052). Among covariates, profound ID (*vs*. mild) was associated with lower PW in Model 1 (B = −1.362, *p* = .035) but was attenuated after adding emotional distress in Model 2. The highest income group (>80000 RMB *vs*. <20000) remained positively associated with PW in Model 2 (B = 1.982, *p* = .012). In addition, only-child status was positively associated with PW in Model 2 (B = 0.817, *p* = .036) (**Table 9B**).

In the SR domain, Model 1 was significant, *F*(15, 201) = 4.892, *p* <.001 (R² = .267; adjusted R² = .213). Adding anxiety and depression significantly improved model fit (ΔR² = .053; *F*-change(2, 199) = 7.720, *p* <.001), and Model 2 remained significant, *F*(17, 199) = 5.514, *p* <.001 (R² = .320; adjusted R² = .262). In the final model, both anxiety (B = −0.116, β = −0.174, *p* = .011) and depression (B = −0.077, β = −0.131, *p* = .048) were significant negative predictors of SR. Several covariates also remained significant in Model 2, including girl (*vs*. boy; B = 2.184, *p* <.001), age 12–14 (*vs*. 6–8; B = 1.787, *p* = .027), and the highest income group (>80000 RMB *vs*. <20000; B = 2.976, *p* = .009), whereas the grandfather (*vs*. father) effect observed in Model 1 was attenuated in Model 2 (**Table 9C**).

In the EN domain, Model 1 was significant, *F*(15, 201) = 4.988, *p* <.001 (R² = .271; adjusted R² = .217). Adding anxiety and depression significantly improved model fit (ΔR² = .038; *F*-change(2, 199) = 5.435, *p* = .005), and Model 2 remained significant, *F*(17, 199) = 5.235, *p* <.001 (R² = .309; adjusted R² = .250). In the final model, depression was a significant negative predictor of EN (B = −0.092, β = −0.167, *p* = .013), whereas anxiety was not significant (*p* = .233). Child age remained positively associated with EN, with higher scores for ages 12–14 (*vs*. 6–8; B = 3.098, *p* <.001) and 15–16 (*vs*. 6–8; B = 2.332, *p* = .009) (**Table 9D**).

## 4 Discussion

This study examined the QOL of primary caregivers of children with ID and the factors associated with it. Compared with caregivers of children without ID, caregivers of children with ID reported significantly lower WHOQOL-BREF scores across all domains and higher levels of anxiety and depression. In bivariate analyses, caregiver QOL was associated with caregiver kinship, child gender, only-child status, and family structure. Child age and family income were positively correlated with QOL, while degree of ID, anxiety, and depression were negatively correlated with QOL. These findings emphasize the critical role of caregiver-related and child-related factors in influencing caregiver QOL.

Given the cross-sectional design, these findings should be interpreted as associations rather than evidence of causal relationships. The negative correlations with anxiety and depression underscore the importance of addressing emotional well-being in caregivers to improve their quality of life. Similarly, the significant impact of family structure and socio-economic factors suggests that interventions aimed at reducing caregiver stress and enhancing social support networks may play a crucial role in improving QOL in this population. Further longitudinal research is needed to explore the causal pathways and the long-term effects of these factors.

### 4.1 Current status of QOL

Caregivers of children with ID scored lower across all WHOQOL-BREF domains than the comparison group, consistent with prior work (37). Among domains, PW was highest and SR was lowest, followed by PC and EN.

Lower PC scores suggest substantial physical and physiological strain. Prolonged caregiving may contribute to fatigue, sleep disturbance, and reduced health maintenance behaviors (38). Many caregivers may deprioritize exercise, rest, and self-care, which could further relate to poorer physical QOL.

In the PW domain, caregivers may still derive meaning and resilience from caregiving roles (39, 40). However, persistent behavioral challenges, financial strain, and chronic stress may erode psychological well-being over time (9, 10, 12), contributing to increased parenting stress and poorer mental health (41).

Low SR scores indicate limited social participation and support. Consistent with prior studies, social and professional support appear important for maintaining caregiver QOL, whereas limited accessible resources and social isolation may exacerbate caregiver stress (42, 43).

The EN domain reflects perceived environmental resources, including social services, housing conditions, and access to facilities. Lower EN scores, consistent with prior findings (44), suggest that environmental conditions and service accessibility may be inadequate for many families, potentially constraining daily functioning and overall satisfaction.

Notably, the between-group difference in the physical domain showed an exceptionally large effect size. This magnitude should be interpreted cautiously, as effect sizes of this scale are uncommon in psychosocial comparative studies and may reflect not only caregiving-related burden but also structural differences between the hospital-recruited ID caregiver group and the online-recruited comparison group. For example, unmeasured differences in baseline health status, service utilization, or participation characteristics could have contributed to the observed physical-domain gap. In addition, the WHOQOL-BREF physical domain may partly capture baseline health differences that are not attributable to caregiving burden per se. Therefore, we interpret these results as associative rather than causal and emphasize the need for future studies using matched sampling strategies or longitudinal designs to better disentangle caregiving effects from sample-composition effects.

### 4.2 Anxiety and depression among caregivers

Caregivers of children with ID exhibited markedly higher levels of anxiety and depression than caregivers of children without ID. Using the HAMA and HAMD-17 thresholds, 37.3% of caregivers of children with ID met criteria for clinically relevant anxiety (HAMA ≥ 14), and 53.0% met criteria for clinically relevant depressive symptoms (HAMD-17 ≥ 7), compared with 13.4% and 12.8% in the comparison group. These rates exceed those reported in a meta-analysis of comparative studies of maternal depression in developmental disabilities (45). Emotional distress in caregivers may arise from financial strain, demanding care routines, stigma, and concerns about the child’s future (11). Persistent uncertainty and limited improvement in rehabilitation outcomes may also contribute to hopelessness and chronic stress, potentially explaining the elevated levels of anxiety and depression observed in this study.

### 4.3 Factors associated with QOL

#### 4.3.1 Caregiver kinship

Caregiver kinship was associated with SR scores in partially adjusted models, with fathers reporting higher SR QOL than grandfathers, consistent with prior findings (13). However, this association was attenuated after adjusting for anxiety and depression, suggesting that differences by kinship may be partly explained by variations in emotional distress rather than kinship status per se.

#### 4.3.2 Child gender

Caregivers of boys reported lower QOL than caregivers of girls, consistent with prior work (46). Cultural expectations for sons may increase stress when boys have ID, and boys’ behavioral challenges may reduce caregivers’ social participation and perceived support.

From a family systems perspective, child gender may interact with parenting expectations and family dynamics, shaping caregivers’ emotional responses and coping strategies. In contexts where boys are expected to be “strong” or more independent, caregiving demands associated with ID may create additional discrepancy between expectations and lived experience, potentially increasing caregiver strain.

#### 4.3.3 Child age

Caregivers of older children (12–16 years) showed better QOL than those caring for younger children (6–11 years), consistent with previous research (47). Over time, families may adapt and develop more effective coping strategies, and children’s increasing functional skills and school/community integration may reduce daily caregiving burden.

#### 4.3.4 Only-child status

In bivariate analyses, caregivers of only children showed lower PW, SR, and EN scores than caregivers of children with siblings. However, in the multivariable models, only-child status was independently associated with PW, and the direction of association indicated higher PW among caregivers of only children after adjustment. This pattern suggests that the relationship between family composition and caregiver QOL may be more complex than suggested by unadjusted comparisons. In China, high expectations for only children may intensify distress when disabilities occur; however, concentrated parental investment and clearer caregiving role allocation in one-child families may also facilitate psychological adaptation under certain conditions (48). This interpretation aligns with stress-buffering perspectives in which family resources and support may mitigate the adverse effects of stressors (49).

#### 4.3.5 Degree of ID

In partially adjusted models, profound ID (*vs*. mild) was associated with lower psychological well-being, consistent with prior work suggesting that greater impairment levels may increase caregiving burden (15). However, this association was attenuated after emotional distress variables were included in the final model. This pattern suggests that the relationship between severity of ID and caregiver QOL may be partly accounted for by caregivers’ anxiety and depressive symptoms rather than impairment severity alone.

#### 4.3.6 Family income

Income was positively associated with QOL in selected domains. In the final multivariable models, caregivers in the highest income group reported higher psychological well-being and social relationship scores compared with those in the lowest income group. However, income was not independently associated with physical capacity or environmental QOL after adjustment. Higher-income families can typically access better healthcare, education, and living conditions, which may particularly enhance psychological and social aspects of well-being. Conversely, low-income families may experience compounded stressors. Consistent with cross-national evidence that social support systems may modulate these effects (50), strengthening local service provision and affordability may be particularly relevant in the Chinese context.

#### 4.3.7 Family structure

Family structure was associated with QOL across domains. Supportive relationships in nuclear and stem families may facilitate shared caregiving and enhance well-being, whereas intergenerational and single-parent families may experience reduced cohesion, higher conflict, or limited resources, contributing to poorer caregiver QOL (51).

### 4.4 Anxiety and depression in multivariable models

In multivariable analyses, anxiety and depression explained additional variance in WHOQOL-BREF domains beyond sociodemographic covariates, but their independent associations differed by domain. Anxiety uniquely predicted lower PW and SR scores, whereas depression independently predicted lower EN and SR scores. In contrast, neither anxiety nor depression showed an independent association with PC after adjustment, despite a significant overall increment in explained variance when both symptoms were added (ΔR² = .034–.053). This pattern suggests that emotional distress may be more proximally linked to subjective psychological and social functioning and perceived environmental resources than to physical capacity once background characteristics are taken into account.

Importantly, the incremental variance explained by anxiety and depression, while statistically significant, was modest (ΔR² = .034–.053), indicating that caregiver QOL is shaped by multiple determinants beyond emotional distress alone. Clinically, this finding does not imply that anxiety and depression are the dominant drivers of QOL; rather, they represent potentially modifiable targets that add measurable explanatory value even after accounting for child and family characteristics.

These findings support the clinical relevance of screening for emotional distress and providing family-centered psychosocial support. Interventions that strengthen coping resources and social support may reduce distress and improve caregiver well-being, consistent with stress-buffering models (49). Given the cross-sectional design, these associations should not be interpreted as causal effects; longitudinal research is needed to clarify temporal pathways and identify potential mediators (e.g., perceived social support, objective caregiver burden, and child behavioral comorbidities).

## 5 Conclusion

In conclusion, this study underscores the significant emotional and QOL challenges faced by primary caregivers of children with ID in China. Caregivers of children with ID reported notably lower QOL across all domains, alongside higher levels of anxiety and depression, when compared to caregivers of children without ID. Sociodemographic factors, such as caregiver kinship, child characteristics, and family income, were identified as key influences on caregiver QOL. Furthermore, emotional distress—specifically anxiety and depression—was found to significantly impact caregivers’ QOL, with domain-specific effects observed. These findings emphasize the need for targeted, family-centered interventions that address both emotional well-being and practical support, aiming to improve the overall quality of life for caregivers. Given the negative emotional toll on caregivers, future research should focus on longitudinal studies to explore causal relationships and the long-term effects of emotional distress on caregiver well-being and family dynamics.

## 6 Limitations and future research

While this study provides valuable insights into caregiver QOL, several limitations should be considered. First, data were collected from only one primary caregiver per family (most commonly the mother), which may not fully represent the perspectives of other family members. Because primary caregivers often experience greater stress exposure and health impacts than other caregivers, future studies should include multiple family caregivers to better capture household-level caregiving dynamics and whole-family well-being (52). This multi-informant approach may also help identify how caregiving responsibilities and coping resources are distributed across family members.

Second, recruitment from different settings (hospital-based *vs*. online) may have introduced selection bias related to service access, socioeconomic status, baseline health, and willingness to participate. Although no significant sociodemographic differences were observed, residual confounding due to unmeasured factors (e.g., health status, child service utilization, and caregiver help-seeking) cannot be ruled out. Future studies should consider matched sampling, propensity score approaches, or recruitment from comparable settings to improve comparability between groups.

Third, measurement heterogeneity may have introduced method-related variance, as anxiety and depression were assessed using clinician-rated instruments (HAMA/HAMD-17) whereas QOL was assessed via self-report (WHOQOL-BREF). Future studies could consider using harmonized informant approaches (e.g., combining self-report symptom scales with clinician-rated measures) and explicitly evaluating potential method effects (53). In addition, formal inter-rater reliability indices (e.g., intraclass correlation coefficients) were not computed for HAMA/HAMD-17 ratings in this dataset; future work should quantify inter-rater reliability to strengthen measurement precision.

Fourth, the cross-sectional design precludes causal inference and limits conclusions about temporal ordering. Longitudinal designs are needed to clarify whether emotional distress precedes changes in QOL, whether QOL declines contribute to emotional distress, or whether both are driven by upstream factors (e.g., child behavioral comorbidities and caregiving burden). Prospective follow-up would also allow testing of potential mediators and moderators, such as social support and family functioning.

Fifth, although sensitivity analyses supported the stability of key inferences, multicollinearity and conceptual overlap between family structure and caregiver kinship limited their simultaneous inclusion in the same regression models. Future research could refine measurement of family-structural indicators (e.g., household composition and caregiving role allocation) or use analytic approaches designed to handle correlated predictors (e.g., penalized regression), to clarify the unique contribution of each construct.

Finally, several potentially important variables were not assessed, including perceived social support, objective caregiving burden, and child behavioral comorbidities, which prior literature indicates are central to caregiver well-being and QOL. Future studies should incorporate these factors to provide a more comprehensive explanatory model and to identify targets for intervention (6).

Overall, future research should employ longitudinal and multi-informant designs, incorporate broader psychosocial and child-related variables, and use improved sampling and measurement strategies to strengthen causal inference and guide family-centered interventions for caregivers of children with ID.

## Data Availability

The original contributions presented in the study are included in the article/supplementary material. Further inquiries can be directed to the corresponding author.
